# P01 Development of a toolkit enabling schools and colleges to confidently support children and young people with Juvenile Idiopathic Arthritis

**DOI:** 10.1093/rap/rkac067.001

**Published:** 2022-09-28

**Authors:** Richard Beesley

**Affiliations:** Juvenile Arthritis Research, Tonbridge, United Kingdom; European Network for Children with Arthritis, Geneva, Switzerland

## Abstract

**Introduction/Background:**

Children and Young People (CYP) spend a considerable amount of time at school, college or other educational settings. Whilst most CYP with Juvenile Idiopathic Arthritis (JIA) are able to access education, many require adaptations, awareness of their needs or specific support to enable them to fully engage in learning. Families of CYP with JIA have reported a lack of awareness and understanding and requested resources to enable schools to support their children in school. The aim of this work was to develop a School Toolkit to allow teachers and other school staff to confidently support CYP with JIA.

**Description/Method:**

Using a network of parents of CYP with JIA, UK-based charity Juvenile Arthritis Research developed a series of resources aimed at schools, colleges, pre-schools and other educational settings. Given awareness of childhood arthritis is low, the Toolkit includes awareness-raising resources, aimed both at staff and all families connected to the school. Increased awareness can help improve diagnosis times and timely access to treatment in those not yet diagnosed. In addition, the Toolkit contains information about what JIA is, resources for schools to provide targeted interventions, and information on how schools can successfully support CYP with JIA. Finally, each Toolkit contains both a presentation for explaining arthritis to children in a classroom or group assembly setting, and a training presentation for delivery to staff, as well as digital copies of key materials and awareness-raising resources for distribution to parents and families.

**Discussion/Results:**

In the week after launching the new School Toolkit, over 20 kits were sent to schools in the UK, potentially providing information that arthritis affects children to over 20,000 families with a further 35 Toolkits in the subsequent month.

Schools have advised they are using the resources in their settings, including giving lessons to children to explain what arthritis is, putting up posters highlighting the key signs and symptoms of JIA, and utilising the staff training resources. Feedback from schools was entirely positive, with one teacher reporting “Wow- it's fantastic, the student presentation is particularly impressive with how it translates all the key information in a child friendly manner.”

**Key learning points/Conclusion:**

The development and supply of a Toolkit specifically for use in schools and colleges has helped raise awareness that children get arthritis and provided resources to train and support school staff enabling them to confidently support CYP with JIA. Utilising the experience and skills of parents and teachers in the development of the Toolkit has ensured all resources are relevant and address the needs of both families and schools. Toolkits can be requested free of charge by schools in the UK from http://www.jarproject.org/toolkit

**Acknowledgements:**

Thanks to the children, young people, families and schools involved in the development of this vital resource.

